# Effect of Mechanical Polishing on Rice Flavor: Comparison and Exploration of Key Aroma Characteristics Components

**DOI:** 10.3390/foods15122205

**Published:** 2026-06-18

**Authors:** Shan Dong, Lele Lu, Li Hou, Wentong Wu, Lidong Wang, Changsheng Li, Changyuan Wang

**Affiliations:** 1 College of Food Science, Heilongjiang Bayi Agricultural University, Daqing 163319, China; dongshan0731@163.com (S.D.); lulele0510@163.com (L.L.); hl1660783228@163.com (L.H.); wwt0426459@163.com (W.W.); wanglidong-521@163.com (L.W.); 2 National Engineering and Technology Research Center for Coarse Grains, Daqing 163319, China

**Keywords:** rice, polishing, volatile flavor compounds, HS-SPME-GC-MS, aroma

## Abstract

Polishing enhances the appearance and market competitiveness of rice. To better understand the effect of polishing on rice flavor, volatile flavor compounds in polished rice (PR), unpolished rice (UR), cooked polished rice (CPR), and cooked unpolished rice (CUR) were examined using headspace solid-phase microextraction coupled with gas chromatography–mass spectrometry (HS-SPME-GC-MS). The results revealed fourteen volatile flavor compounds displayed significant differences in abundance, with eight of these compounds potentially contributing to the overall flavor profile based on their volatility and reported odor characteristics. Among these compounds, only eicosane and hexanal were detected in uncooked rice, whereas acetophenone, hexadecanol, dodecane, and octadecane were unique to CUR. Four compounds were associated with aroma notes reminiscent of flowers, wax, and almond, among others. However, nonanal and nerol were common in both cooked rice samples, and they may contribute to a sweet-like aroma in cooked rice. These findings illuminate the changes in volatile composition, offer insights to prevent over-polishing, and inspire further research toward producing rice with potentially improved aroma profiles.

## 1. Introduction

Rice (*Oryza sativa* L.) is one of the most essential food crops worldwide. In China, rice constitutes over 40% of all food crops, and serves as a staple food for more than 60% of the population [1]. The process of husking paddy yields brown rice, which is rich in dietary fiber, proteins, vitamins, and other nutrients. However, brown rice is not widely accepted as an ideal staple food owing to its coarse texture and cooking difficulties [2]. Therefore, brown rice typically undergoes extensive processing to produce white rice for consumption. Although unpolished rice (UR) produced by a rice milling machine exhibits superior taste qualities, its surface is covered with substantial amounts of broken bran, aleurone grains, and other materials. These residues not only diminish the appearance quality of rice but also significantly reduce its shelf life [3]. Therefore, industrial rice production requires polishing equipment to refine rice [4]. Polishing, a common step in rice processing, enhances grain brightness, improves color, and elevates overall appearance quality. However, this process leads to the loss of numerous nutrients and flavor compounds, partially diminishing the aroma of rice. Studies have shown that polished rice (PR) loses certain flavor compounds, including those associated with sweet and umami-like aroma notes, compared to UR [5]. To date, researchers have identified more than 500 volatile aromatic compounds in various scented and non-scented rice cultivars. These compounds primarily consist of alcohols, esters, aldehydes, ketones, hydrocarbons, furans, and compounds containing benzene, sulfur, chlorine, and nitrogen rings [6]. These aroma compounds impart unique flavors to rice, such as blandness, burning, buttery, biscuit, nutty, popcorn, and flower scents, all of which enhance consumer acceptance [1].

The aroma of rice varies significantly across different varieties and origins, contributing to its economic value because of its characteristic aroma profile [1]. Cooked rice, a staple food prepared via hydrothermal cooking, exhibits a volatile profile distinctly different from that of uncooked rice. This variation arises from multiple reactions that occur during cooking, including enzymatic and non-enzymatic hydrolysis, oxidation, Maillard reaction, and thermal decomposition. Moreover, the intricate matrix of rice involves complex interactions between proteins, starch, fat, and aroma compounds, which affect both aroma formation and release. Ma et al. [7] revealed that the separation of starch and lipids during cooking facilitates the formation of lipid-based flavor compounds. Wang et al. [8] also demonstrated that amylose and flavor substances can form thermally stable structures, allowing for the release of these compounds upon reheating. Although the cooking process significantly affects rice flavor, the loss of rice constituents during milling and polishing also plays a crucial role in determining the final flavor profile. Zeng et al. [9] identified esters, alkanes, alcohols, ketones, aldehydes, and acids as the primary volatile compounds in rice bran, with flavor compounds constituting 80% of the total aromatic components. Similarly, Yan et al. [10] observed that fresh rice bran retained an abundant concentration of aldehydes and alkanes, preserving its aroma. Although numerous studies have confirmed flavor changes in rice bran, research on flavor loss during processing remains limited. Consequently, further investigation into the relationship between processing methods and flavor compounds is necessary to optimize the efficient use of byproducts of rice processing.

Despite the benefits of rice polishing in terms of improving the appearance quality, it is detrimental to the retention of both nutritional and flavor properties. Concerns persist that rice polishing represents an unnecessary expenditure on resources and energy. To address these concerns, this study investigated the flavor profile of rice before and after polishing, as well as after cooking. Unlike previous work that focused either on raw milled rice or on bran volatiles separately, we systematically compared the volatile composition of unpolished rice (UR) and polished rice (PR), and further extended the comparison to cooked UR (CUR) and cooked PR (CPR). This allowed us to distinguish among compounds inherently present in raw rice, those lost or generated during polishing, and those produced during cooking. This comprehensive comparison clarifies the direct impact of polishing on rice flavor, supports the minimization of excessive polishing, conserves resources and energy, and generates insights into enhancing the aromatic quality of rice.

## 2. Materials and Methods

### 2.1. Preparation of UR and PR

Daohuaxiang paddy rice was provided by Heilongjiang Beidahuang Rice Industry Group Wuchang Co., Ltd. (Harbin, China). The paddy rice was harvested in October 2023, dried to a moisture content of 12–14%, and stored in a warehouse at 10–15 °C, where the humidity was maintained at 60–70%. Three separate batches of rice were used. The rice was de-husked using a husker (Model THU35C, Satake Manufacturing Suzhou Co., Ltd., Suzhou, China), and any remaining impurities were manually removed to obtain clean brown rice. The prepared brown rice was stored in a controlled warehouse at 10–15 °C for future research. A rice mill with a silicon carbide roller (Model TM05C, Satake Manufacturing Suzhou Co., Ltd., Suzhou, China) was used to mill brown rice. A 150 g sample of brown rice, with a moisture content of 14.5%, was weighed and introduced into the feed port of a rice mill (silicon carbide roller diameter of 30 Φ). The milling process was performed for 100 s to produce the UR. Subsequently, PR was obtained by polishing the UR using a rice polisher (Model LTJM-2099, Shanghai Precision Instrument Co., Ltd., Shanghai, China). 

### 2.2. Preparation of CUR and CPR

UR and PR were steamed in an electric pressure cooker (MY-S572N; Midea Group Co., Ltd. Guangzhou, China), with a rice-to-water ratio of 1:1.3. Steamed UR and steamed PR were designated as CUR and CPR, respectively. Following steaming, rice samples were frozen in liquid nitrogen and freeze-dried using a freeze dryer (TF-FD-27S, Shanghai Tianfeng Industrial Co., Ltd. Shanghai, China). All rice samples, including UR, PR, CUR, and CPR, were stored at −80 °C [11]. The three replicates were independent biological replicates; three separate batches were prepared for each sample type and analyzed independently.

### 2.3. Image Analysis and Scanning Electron Microscope (SEM)

Rice samples were stained with eosin Y and methylene blue. The rice was immersed in the dye solution for 3 min, after which the dye was removed, and the samples were rinsed with 80% ethanol solution for 1 min. This rinsing process was repeated three times. The stained rice was then placed on a filter paper to remove excess moisture before use. Pre- and post-staining images of UR, PR, CUR, and CPR were captured using a rice imaging instrument (JWCT 12; Beijing Dongfu Jiuheng Instrument Technology Co., Ltd., Beijing, China). For SEM imaging, UR and PR samples were mounted on double-coated conductive tape (3M, St. Paul, MN, USA) and subjected to gold coating at 10 mA for 5 min. SEM images were acquired using a scanning electron microscope (EVO18, Carl Zeiss AG, Oberkochen, Germany) operating at 5.0 kV [12].

### 2.4. Volatile Compound Analysis by HS-SPME-GC/MS

#### 2.4.1. Extraction of Samples

As described by Li et al. [13], the aroma compounds in the rice samples were extracted by HS-SPME. A 50/30 µm DVB/CAR/PDMS solid-phase microextraction fiber (Supelco, Inc., Bellefonte, PA, USA) was used. 3 g of rice sample powder, 5 mL distilled water, and 1.8 g NaCl were weighed into a 20 mL headspace vial. Subsequently, 5 µL of 2,4,6-trimethylpyridine (10 µL dissolved in 40 mL of methanol) was added as internal standard. The vial was then incubated with agitation at 70 °C for 20 min, followed by exposure of the fiber at 70 °C for 45 min (MPS 2XL, Gerstel GmbH & Co. KG, Mülheim an der Ruhr, Germany).

#### 2.4.2. GC-MS Analysis

The analysis was performed using an Agilent 8890 gas chromatograph coupled with an Agilent 7000D mass spectrometer (Agilent, Santa Clara, CA, USA), equipped with a Gerstel MPS 2XL autosampler for SPME injection (Gerstel GmbH & Co. KG, Mülheim an der Ruhr, Germany). Separation was achieved on a VF-WAXms capillary column (25 m × 0.25 mm × 0.2 µm). Helium (99.999%) was used as the carrier gas at a constant flow rate of 2 mL/min. The SPME fiber was desorbed in the GC injection port at 250 °C for 5 min in split mode with a ratio of 10:1. The injector temperature was set at 250 °C. The oven temperature program was as follows: initial temperature 40 °C held for 2 min, then increased to 100 °C at 5 °C/min, then to 230 °C at 15 °C/min, held for 5 min, and finally held at 230 °C for an additional 2 min. The mass spectrometer was operated in electron impact (EI) mode at 70 eV. The ion source temperature was 230 °C, and the quadrupole temperature was 150 °C. The transfer line temperature was 310 °C. Full scan mode was employed over a mass range of *m*/*z* 30–1000 with a scan rate of 3.2 scans/s.

#### 2.4.3. Data Preprocessing and Annotation

Raw data acquired from GC/MS mass spectrometer detection were preprocessed using MassHunter workstation Quantitative Analysis software (version v10.0.707.0), resulting in the export of a three-dimensional data matrix in the CSV format. This matrix encompasses the sample information, metabolite names, and mass spectral response intensities. The data matrix was refined by eliminating internal standard peaks and known false-positive peaks (e.g., noise, column bleed, and derivatized reagent peaks), followed by de-redundancy and peak pooling. Metabolite identification was conducted through database searches, primarily utilizing public databases such as NIST (version 2017), FiehnLib (version 2013), and MS-DIAL (version 2021). Only matches with a similarity score > 800 (NIST) or equivalent quality criteria were accepted.

The resulting data matrix was uploaded to the Majorbio Cloud platform (https://cloud.majorbio.com, accessed on 30 December 2024) for further analyses. The preprocessing involved retaining at least 80% of the metabolic features detected in each sample set. For samples with metabolite levels below the quantification limit, the minimum metabolite values were estimated and each metabolic signature was normalized to the total sum of peak intensities within the sample. To address potential errors from sample preparation and instrument instability, the sample mass spectrometry peak response intensities were subjected to sum normalization. Quality control (QC) sample variables with a relative standard deviation (RSD) exceeding 30% were excluded, and the data were log10-transformed and used to yield the final matrix for subsequent analysis. Principal component analysis (PCA) and partial least squares discriminant analysis (PLS-DA) were employed to assess model stability, and significantly different metabolites were identified.

Differential metabolites identified groups were mapped to biochemical pathways through metabolic enrichment and pathway analysis, utilizing the KEGG database (http://www.genome.jp/kegg/, accessed on 30 December 2024). Metabolites were categorized based on their involvement in pathways or their functional roles. Enrichment analysis was conducted to evaluate the presence of metabolite groups within the function nodes, expanding from single-metabolite annotation to group-based annotation. The Python 3.12 package “scipy.stats” (https://docs.scipy.org/doc/scipy/, accessed on 30 December 2024) facilitated enrichment analysis to determine the most relevant biological pathways for experimental treatments.

### 2.5. Statistical Analysis

The data obtained from triplicate measurements were subjected to statistical analyses. Statistical analyses were conducted using SPSS (version 22; IBM, Armonk City, New York, NY, USA) and Origin software (version 9.1; OriginLab, Northampton, MA, USA). Results were presented as mean ± standard deviation, with statistical significance set at a 95% confidence level (*p* < 0.05) and assessed using one-way ANOVA (Duncan).

## 3. Results

### 3.1. Changes in Appearance of UR, PR, CUR, and CPR

The brown rice bran layer comprises three primary layers: pericarp, testa, and nucellar layer. Beneath the bran is the aleurone layer, which consists of aleurone cells, followed by the endosperm structure containing starch, protein, and various other components [14]. The rice-milling process primarily involves crushing the bran layer. Nevertheless, the presence of the residual aleurone layer and bran powder negatively affected the visual quality of the rice. Therefore, a polishing step is employed in the final stage of milling to enhance the appearance of the rice [15].

**Figure 1:**
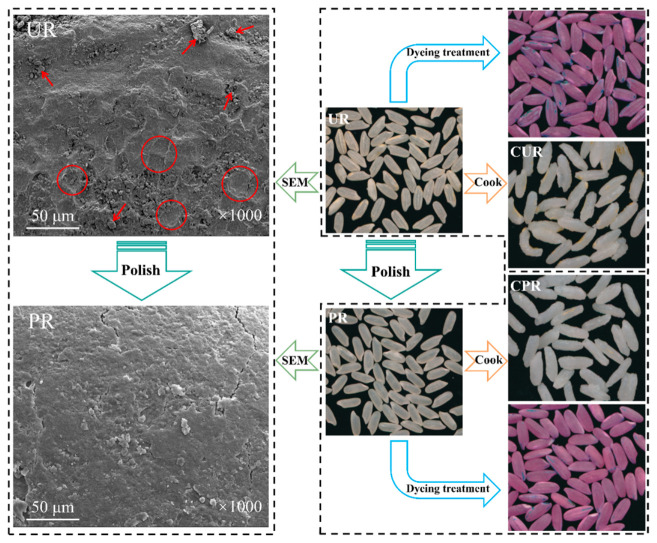
Changes in the rice before and after polishing. (Broken aleurone cell cavity structure shown in red circles; the red arrow shows the bran powder).

The morphological changes in the rice before and after polishing are shown in Figure 1. The UR exhibited a light-yellow hue, with its surface characterized by long strips and flaky structures, resulting from incomplete milling of the bran [16]. Rice products processed in this manner have an inferior appearance and are generally unacceptable to consumers. PR has a white coloration with a relatively smooth surface and is devoid of excess cortical residue. The post-polishing appearance of rice is white, lustrous, and uniformly colored, conferring superior appearance quality and increased consumer acceptability, thus becoming the predominant product in the rice market [17]. Upon staining, the bran layer and germ manifested a blue coloration, whereas the endosperm assumed a purple-red hue, attributable to variations in the affinities of the rice bran layer, endosperm, and germ for eosin Y and methylene blue. Discontinuous long strip structures persisted on the UR surface, and although PR also exhibited a residual bran, they were present in lesser quantities on the PR surface. The overall color distribution of the PR surface was uniform, potentially because of the removal of bran powder and aleurone cells from the UR surface during polishing, allowing for complete interaction between the endosperm and eosin Y, resulting in visual differences after staining [14]. A significant difference was observed between UR and PR after cooking, with more cortical residue visible on the surface of CUR, rendering the rice grains relatively dull. Although the surface of the CPR also exhibited some residual bran, its rice grains were white and shiny, providing a better visual experience. Electron microscopic examination of UR and PR revealed that the UR surface was covered with granular bran and spherical aleurone grains. Concurrently, a regular polygon structure is observed on the surface, representing the cavity structure formed after the disruption of aleurone cells [18]. In contrast, the polishing process eliminated all aleurone particles and bran powder, resulting in a relatively flat and smooth PR surface with no observable residual aleurone cell structure. The aforementioned observations indicate that UR has a higher nutritional value than PR, which also creates favorable conditions for the formation of more flavor compounds in cooked rice [19].

### 3.2. Identification and Classification of Volatile Compounds of UR, PR, CUR, and CPR

The analysis of volatile compounds is crucial for understanding the aromatic profiles of rice. In this study, 17 volatile compounds were detected in UR, PR, CUR, and CPR, encompassing eight distinct categories: 2 aldehydes, 2 alcohol, 1 amine, 1 ether, 2 phenols, 2 ketones, 6 hydrocarbons, and 1 other compounds (Table 1). Odor descriptors source is the Majorbio Cloud platform (https://cloud.majorbio.com). The volatile compounds generated across the different stages of rice processing (unpolished, polished, and cooked) collectively contribute to a wide spectrum of olfactory sensations, including fatty, grassy, fruity, rose, citrus, waxy, floral, vanilla, gasoline, and alkane notes. The intricate rice matrix involves complex interactions between flavor compounds, proteins, starch, and fat, which collectively influence aroma perception [1]. Previous investigations have highlighted the significance of volatile flavor compounds, particularly aldehydes and phenols, in shaping the flavor of rice during cooking. Notably, hexanal, nonanal, vanillin, and nerol have been identified as key contributors to the aroma of cooked rice [20]. The presence of vanillin can be attributed to the thermal decarboxylation of ferulic acid during cooking, as well as enzymatic hydrolysis during storage, potentially accounting for the formation of its isomer, ortho-vanillin [21]. Nonanal and hexanal are likely products of lipid oxidation in rice, imparting their characteristic aroma [6]. Among the identified volatile flavor compounds, six were hydrocarbons, possibly resulting from starch degradation during gelatinization or the oxidative degradation of fatty acids. However, most hydrocarbons are odorless, with only a few exhibiting gasoline-like scents [22]. The formation of these volatile flavor compounds is intricately linked to the degradation of proteins, fatty acids, and starch during polishing and cooking processes. Furthermore, the Maillard and caramelization reactions that occur during cooking positively influence the generation of volatile flavor compounds [7].

### 3.3. Principal Component (PCA) and Partial Least Squares Discriminant Analysis (PLS-DA)

**Figure 2:**
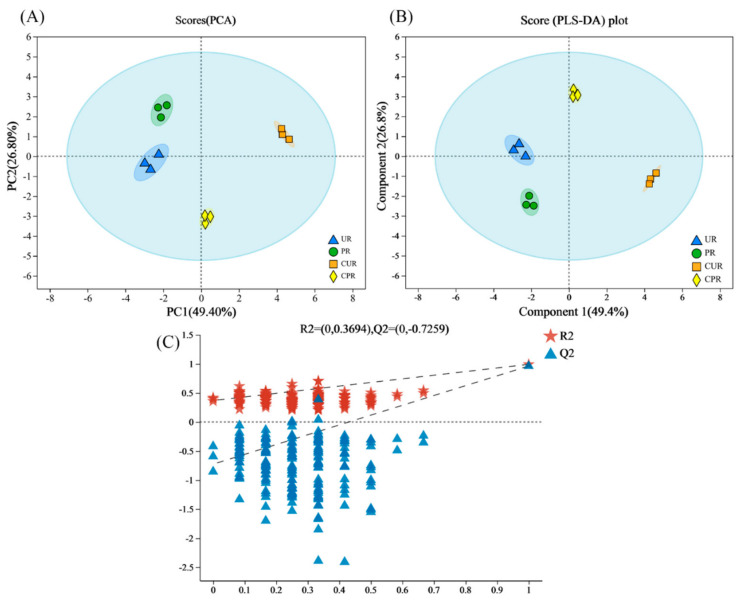
(**A**) Principal component analysis (PCA). (**B**) Partial least squares discriminant analysis (PLS-DA). (**C**) Permutation test using PLS-DA.

The PCA of the samples is shown in Figure 2A, where PC1 and PC2 accounted for 49.40% and 26.80% of the variance, respectively, with an R^2^X value of 0.76 across the sample groups. Despite this analysis, PCA failed to clearly differentiate other metabolites across the various sample groups. Therefore, a PLS-DA model was applied to further characterize metabolite distinctions (Figure 2B). The cumulative Q2Y and Q2 values reached 0.99 and 0.96, respectively, with a negative intercept on the *Y*-axis for the Q2 regression line (−0.7259) following 200 permutation tests (Figure 2C), indicating that the model is free from overfitting and has strong predictive capabilities [23]. Both PCA and PLS-DA revealed a high degree of clustering within each sample group, effectively organizing the samples into four distinct clusters. This clustering suggests that data processing was both reproducible and reliable, while also indicating significant metabolic differences among the four sample groups.

### 3.4. Variation in the Volatile Compounds of UR, PR, CUR, and CPR

**Figure 3:**
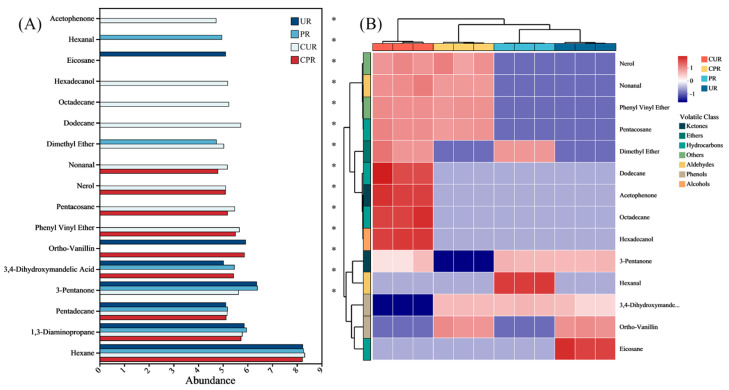
(**A**) Relative abundance diagram of volatile flavor compounds. (**B**) Clustering heat map of volatile flavor compounds. * indicates a statistically significant difference between groups, with *p* ≤ 0.05.

Figure 3A shows the changes in the abundance of volatile flavor compounds in rice before and after polishing and cooking. Polishing and cooking had significant effects on the volatile flavor compounds in rice (*p* < 0.05). Of the 17 identified volatile compounds, 14 displayed significant changes following treatment, whereas three remained unchanged. Unique volatile compounds were identified across the rice samples, and four distinct volatile compounds, eicosane, ortho-vanillin, 3,4-dihydroxymandelic acid, and 3-pentanone, were detected in the UR. Hexanal, dimethyl ether, 3,4-dihydroxymandelic acid, and 3-pentanone were observed in PR. CUR samples revealed 10 unique volatile compounds, including acetophenone, hexadecanol, octadecane, dodecane, dimethyl ether, nonanal, nerol, pentacosane, phenyl vinyl ether, and 3-pentanone. Six different volatile compounds, nonanal, nerol, pentacosane, phenyl vinyl ether, ortho-vanillin, and 3,4-dihydroxymandelic acid, were detected in the CPR.

To elucidate the differences in volatile compounds among the rice samples, 14 significantly different volatile compounds (from the initial 17) were selected for clustering analysis. The clustering heat map of volatile flavor compounds (Figure 3B) showed significant shifts in the relative expression of these differential compounds following polishing and cooking. These compounds are categorized into seven classes: ketones, ethers, hydrocarbons, aldehydes, phenols, alcohols, and others. In UR, compared to PR, ortho-vanillin and eicosane volatiles were upregulated, while dimethyl ether and hexanal were downregulated. Comparing CUR with CPR, dimethyl ether, dodecane, acetophenone, octadecane, hexadecanol, and 3-pentanone were upregulated, whereas 3,4-dihydroxymandelic acid and ortho-vanillin were downregulated. Notably, dodecane, acetophenone, octadecane, and hexadecanol exhibited the most substantial upregulation in cooked rice. In contrast, raw rice exhibited a smaller magnitude of change, likely because of its lower initial volatile flavor compound content and minimal influence of structural changes in the aleurone layer [13]. Compared to raw rice, the Maillard and caramelization reactions that occur during cooking significantly enhance the enrichment of volatile flavor compounds and aroma of CUR [7]. However, the mechanism underlying the enrichment of key volatile flavor compounds in UR and CUR warrants further investigation.

### 3.5. Contribution to Aroma of Key Volatile Flavor Compounds

**Figure 4:**
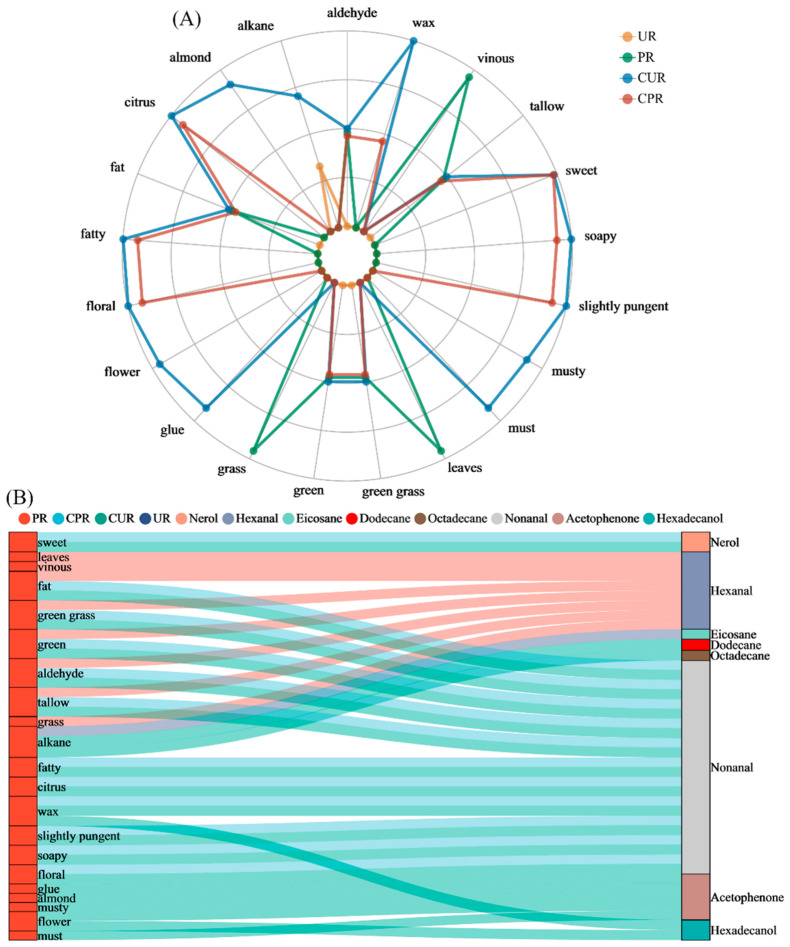
(**A**) Radar map of sensory flavor compound content. (**B**) Sensory flavor characteristics and flavor compound Sankey map.

Odor, a fundamental physical property of matter, arises from the volatilization of aromatic compounds. These aroma molecules travel through the olfactory system to produce a sensory perception [13]. The diverse olfactory profiles of volatile substances, coupled with their relative abundances, shape the overall aroma characteristics of foods [24]. Figure 4A demonstrates the significant impact of the polishing and cooking processes on the flavor profile of rice. The analysis revealed 21 distinct odors in rice, including floral, grassy, green, and fatty notes. Polishing led to a marked increase in the alkane odor in raw rice, while simultaneously diminishing aldehyde, vinous, tallow, leafy, green, grassy, and fatty odors. The aroma profile of cooked rice was notably altered compared with that of raw rice. Studies have indicated that raw rice tends to contain more volatile compounds with minimal aromatic characteristics, whereas cooked rice typically possesses a richer array of aromatic compounds [25,26]. In CPR, aromas such as vinous, leafy, and grassy tones significantly diminished, whereas the presence of waxy, sweet, soapy, slightly pungent, floral, and fatty notes substantially increased. Although UR exhibited a limited aroma profile, the odor intensity of CUR was significantly enhanced upon steaming. Beyond the intensification of the alkane odor, CUR was enriched in almond, glue, floral, and other odors that were absent in CPR, suggesting potential contributions to its sensory and edible qualities. The Sankey map in Figure 4B illustrates the relationship between sensory characteristics and specific flavor compounds. Eight volatile flavor compounds play crucial roles in the rice aroma: nonanal, hexanal, nerol, eicosane, hexadecanol, acetophenone, dodecane, and octadecane. Eicosane in UR may be associated with alkane-like odor notes, while hexanal in PR has been reported to contribute to leafy, vinous, aldehyde, tallow, grassy, and green odors in previous studies [27]. Similarly, nonanal and nerol are known from the literature to be linked to sweet, fatty, green, tallow, alkane, citrus, waxy, soapy, and aldehyde profiles [28,29]; therefore, their higher abundance in CPR and CUR suggests a potential contribution to these aroma attributes. The unique flavors of CUR, such as glue, almond, floral, and alkane notes, were attributed to acetophenone, hexadecanol, dodecane, and octadecane, which contributed to the distinct flavor differences between CUR and CPR.

### 3.6. Analysis of Key Volatile Flavor Compounds Pathways in Polishing and Cooking

**Figure 5:**
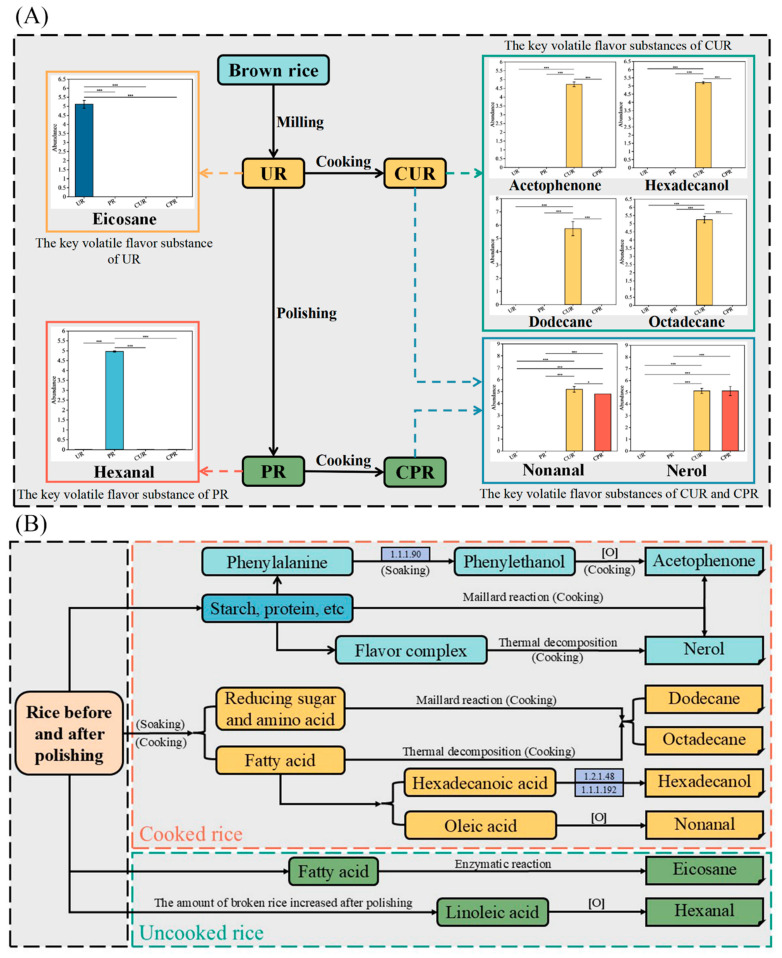
(**A**) Effect of polishing treatment on 8 key volatile flavor substances in rice. (**B**) Possible pathways of rice key differential flavor compounds formation by polishing and cooking. Statistical significance between groups is indicated by asterisks: * for *p* ≤ 0.05and *** for *p* ≤ 0.001.

Processing accuracy plays a vital role in preserving rice flavor. Polishing, a crucial step in rice processing, eliminates numerous nutrients, potentially leading to direct or indirect loss of flavor compounds [15]. A total of 21 unique flavor characteristics were identified in rice (Figure 4A,B). Figure 5A shows that uncooked rice contains two significantly different flavor compounds with unique flavors, whereas cooked rice contains six. Eicosane was detected in UR, whereas hexanal was identified in PR. CUR revealed the presence of four key volatile flavor compounds: acetophenone, hexadecanol, dodecane, and octadecane. The detection of nonanal and nerol in both CUR and CPR suggests their potential role as characteristic flavor compounds in cooked rice [30,31].

Figure 5B outlines the possible formation pathways of these flavor compounds before and after the polishing process. Alkane compounds are recognized as significant aroma contributors in rice. It has been suggested that residual bran and the aleurone layer on the surface of unpolished rice (UR) facilitate lipase retention, which may promote alkane formation. Yan et al. [10] proposed that the presence of lipase in rice bran provides favorable conditions for alkane compound generation. Moreover, the state of rice processing significantly influenced the hexanal content. Yang et al. [32] demonstrated that broken rice contains substantially higher hexanal concentrations than the whole rice. This phenomenon may be attributed to the increased contact area of broken rice exposed to air, which promotes the oxidation of free fatty acids such as linoleic acid to form hexanal [33]. During polishing, rice grains experience prolonged friction and collisions [19]. Additionally, due to the lower structural hardness of the rice endosperm, it has limited resistance to mechanical stress [34]. Therefore, polishing often leads to an increased production of broken rice, and the elevated hexanal levels observed in our polished rice samples may be partly attributed to this increased proportion of broken rice.

The degree of processing significantly influences the nutrient and enzyme contents of UR, resulting in higher levels than those of PR. During rice soaking prior to cooking, endogenous enzymes more readily degrade nutrients such as starch, fat, and protein in UR, yielding free reducing sugars, fatty acids, and amino acids. These degradation products serve as substrates for the Maillard reaction and can undergo further oxidation to generate additional flavor compounds [35]. High-temperature cooking of UR, with its elevated fatty acid content, facilitates an increase in dodecane and octadecane. Although the Maillard reaction mainly produces heterocyclic aroma compounds, it may also participate in cross-reactions with lipid degradation products, potentially affecting the alkane profile. Fatty acid oxidation contributes to the enrichment of alkane compounds and aldehyde formation. Guan and Zhang [1] suggested that nonanal results from high-temperature oxidation of oleic acid in rice. The increased nonanal abundance in UR can be attributed to the rice soaking and cooking processes. Soaking enhances oleic acid formation by increasing endogenous enzyme activity, whereas elevated cooking temperatures accelerate oleic acid oxidation, potentially leading to nonanal enrichment [1,36].

Alcohols, along with aldehydes and alkanes, contribute substantially to the aroma profile of cooked rice [37]. Hexadecanol, detected in CUR, may arise from the thermal degradation of sugars and lipids as well as from enzymatic hydrolysis. Increased enzyme activity during soaking fosters conditions favorable for fatty acid formation [1]. During soaking, hexadecanol may be formed via the activity of long-chain fatty aldehyde dehydrogenase (1.2.1.48) and long-chain alcohol dehydrogenase (1.1.1.192). Concurrently, in UR, aryl-alcohol dehydrogenase (1.1.1.90) activity may be continuously activated, promoting the hydrolysis of phenylalanine and enriching phenylethanol, which can be converted to acetophenone through high-temperature oxidation in subsequent cooking [1]. Simultaneously, macromolecules such as starch and proteins in rice can establish covalent bonds with small molecular flavor compounds, forming complexes. These complexes exhibit extreme instability at elevated temperatures, leading to the liberation of flavor compounds during high-temperature cooking [1]. Dziadas and Jeleń [38] demonstrated that nerol and carbohydrates form complexes via glucoside bonds, which can be disrupted by high-temperature heating, resulting in nerol release. However, during high-temperature cooking, rice components, such as starch and protein, may undergo reactions with various substances, potentially contributing to the enrichment of both acetophenone and nerol [37].

## 4. Conclusions

In modern rice processing, polishing is a critical step that removes bran particles, whitens and brightens rice grains, and enhances the visual quality of rice. However, excessive polishing can lead to significant energy wastage, which may reduce nutrient retention. Therefore, this study investigated the volatile flavor compounds in rice by examining the differences in flavor characteristics between polished and unpolished rice. The findings revealed that the UR surfaces displayed discontinuous long strip structures covered with granular bran and spherical aleurone grains, while the PR surface, with reduced bran, showed enhanced visual quality. Notably, CUR grains appeared dull, whereas CPR grains presented a white and shiny surface, offering a better visual experience. HS-SPME-GC-MS analysis of volatile compounds identified significant variations in the abundance of 14 volatile flavor compounds across UR, PR, CUR, and CPR samples. Eight compounds, eicosane, hexanal, nerol, nonanal, dodecane, octadecane, acetophenone, and hexadecanol, contributed significantly to rice flavor. Specifically, eicosane and hexanal are characteristic compounds of uncooked rice, with hexanal predominating in PR and eicosane predominating in UR. Unique compounds in CUR include acetophenone, hexadecanol, dodecane, and octadecane, while nonanal and nerol are common in cooked rice. The increase in flavor compounds in CUR may result from nutrient and enzyme retention in UR, facilitating enzymatic reactions during soaking and enhancing Maillard reactions during cooking. These findings offer theoretical support for reducing excessive rice polishing, helping conserve resources and energy, and inspiring new research directions to enhance rice aroma. Future studies should focus on comparing aroma variations across different post-polishing rice varieties to establish a more comprehensive theoretical framework for guiding rice production.

## Figures and Tables

**Table 1:** Aromatic characteristics of 17 key volatile compounds.

No.	Volatile Class	Volatile Compounds	Formula	CAS ID	RT (min)	RI	Odor Description
1	Aldehydes	Hexanal	C_6_H_12_O	66-25-1	2.91	1092	Fresh, Green, Fatty, Grassy, Fruity
2		Nonanal	C_9_H_18_O	124-19-6	5.49	1576	Fresh, Green, Rose, Citrusy, Fatty
3	Alcohols	Nerol	C_10_H_18_O	68311-14-8	1.42	867	Rose, Floral, Fruity, Citrusy
4		Hexadecanol	C_16_H_34_O	124-29-8	10.78	1976	Waxy, Floral
5	Amines	1,3-Diaminopropane	C_3_H_10_N_2_	18773-03-0	26.79	3071	/
6	Ethers	Dimethyl Ether	C_2_H_6_O	157621-61-9	2.03	959	Ethereal, Ether-like
7	Phenols	3,4-Dihydroxymandelic Acid	C_8_H_8_O_5_	14883-87-5	7.10	1661	/
8		Ortho-Vanillin	C_8_H_8_O_3_	148-53-8	26.93	3079	Milk, Vanilla
9	Ketones	Acetophenone	C_8_H_8_O	98-86-2	7.70	1688	Floral, Almond, Hawthorn, Acacia
10		3-Pentanone	C_5_H_10_O	96-22-0	26.91	3078	/
11	Hydrocarbons	Hexane	C_6_H_14_	50981-41-4	1.36	858	Gasoline, Alkane
12		Dodecane	C_12_H_26_	112-40-3	3.75	1198	Gasoline, Alkane
13		Eicosane	C_20_H_42_	112-95-8	4.10	1403	Alkane, Waxy
14		Octadecane	C_18_H_38_	593-45-3	5.54	1580	Alkane
15		Pentadecane	C_15_H_32_	629-62-9	7.43	1676	Alkane, Waxy
16		Pentacosane	C_25_H_52_	629-99-2	7.74	1691	/
17	Others	Phenyl Vinyl Ether	C_8_H_8_O	25588-11-8	16.83	2409	/

## Data Availability

The original contributions presented in the study are included in the article; further inquiries can be directed to the corresponding authors.
